# Cluster randomized trials utilizing primary care electronic health records: methodological issues in design, conduct, and analysis (eCRT Study)

**DOI:** 10.1186/1745-6215-15-220

**Published:** 2014-06-11

**Authors:** Martin C Gulliford, Tjeerd P van Staa, Lisa McDermott, Gerard McCann, Judith Charlton, Alex Dregan

**Affiliations:** 1 Department of Primary Care and Public Health Sciences, King’s College London, Capital House, 42 Weston St, London SE1 3QD, UK; 2 NIHR Biomedical Research Centre, Guy’s and St Thomas’ Hospital, Great Maze Pond, London SE1 9RT, UK; 3 Clinical Practice Research Datalink (CPRD), Medicines and Healthcare Products Regulatory Agency, London, UK; 4 Division of Primary Care and Population Sciences, University of Southampton, Southampton General Hospital, Mailpoint 801, South Academic Block, Tremona Road, Southampton SO16 6YD, UK

**Keywords:** Clinical trial, Cluster randomization, Electronic health records, Primary care, Implementation science, Decision support

## Abstract

**Background:**

There is growing interest in conducting clinical and cluster randomized trials through electronic health records. This paper reports on the methodological issues identified during the implementation of two cluster randomized trials using the electronic health records of the Clinical Practice Research Datalink (CPRD).

**Methods:**

Two trials were completed in primary care: one aimed to reduce inappropriate antibiotic prescribing for acute respiratory infection; the other aimed to increase physician adherence with secondary prevention interventions after first stroke. The paper draws on documentary records and trial datasets to report on the methodological experience with respect to research ethics and research governance approval, general practice recruitment and allocation, sample size calculation and power, intervention implementation, and trial analysis.

**Results:**

We obtained research governance approvals from more than 150 primary care organizations in England, Wales, and Scotland. There were 104 CPRD general practices recruited to the antibiotic trial and 106 to the stroke trial, with the target number of practices being recruited within six months. Interventions were installed into practice information systems remotely over the internet. The mean number of participants per practice was 5,588 in the antibiotic trial and 110 in the stroke trial, with the coefficient of variation of practice sizes being 0.53 and 0.56 respectively. Outcome measures showed substantial correlations between the 12 months before, and after intervention, with coefficients ranging from 0.42 for diastolic blood pressure to 0.91 for proportion of consultations with antibiotics prescribed, defining practice and participant eligibility for analysis requires careful consideration.

**Conclusions:**

Cluster randomized trials may be performed efficiently in large samples from UK general practices using the electronic health records of a primary care database. The geographical dispersal of trial sites presents a difficulty for research governance approval and intervention implementation. Pretrial data analyses should inform trial design and analysis plans.

**Trial registration:**

Current Controlled Trials ISRCTN 47558792 and ISRCTN 35701810 (both registered on 17 March 2010).

## Background

Recent developments in electronic health records (EHRs), and their increasing accessibility for health research, have stimulated interest in utilizing EHRs for intervention studies in clinical medicine, public health, and health services research [1]. This approach to trial conduct offers several potential advantages over traditional trials. Use of data routinely collected from EHRs facilitates direct access to large sample sizes and reduces the cost of trial implementation. Trials using EHRs may also have high external validity because of the representativeness of the samples that may be recruited and the use of interventions that are similar to those that may be rolled out into routine service settings. Trials using EHRs generally adopt a pragmatic perspective [2], and are sometimes referred to as ‘point-of-care’ trials [3].

In the UK, the main focus of interest has been in the use of primary care EHRs collected into large data resources such as the Clinical Practice Research Datalink (CPRD) [4]. The CPRD presently includes anonymized electronic health record data for about 7 to 8% of UK family practices with a similar proportion of the UK population as registered patients. General practice data in the CPRD are subject to quality checks and, when the data reaches set research standards, are referred to as ‘up-to-standard’ (UTS). The high quality of CPRD data has been extensively documented [5]. CPRD data are presently being enriched through linkages with other data sources including disease registry data [6,7] and data on hospital utilisation and mortality records.

Our group has recently completed two cluster randomized trials using the CPRD [8,9]. These are among the first cluster randomized trials to be performed exclusively using electronic health records. One trial aimed to improve standards of care for secondary prevention after a stroke; this provided an exemplar of a less frequent long-term condition of public health importance. This will be referred to as the ‘stroke trial’. The second trial aimed to reduce the prescribing of antibiotics to patients presenting with respiratory tract infections in primary care. This provided an exemplar of a common acute condition. The trials’ designs [8,9] and substantive results [10,11] have been reported elsewhere.

In view of the novelty of this approach to conducting cluster trials, we present a narrative account of the experience of cluster trial performance using EHRs. This paper aims to report on methodological issues in the design, conduct, and analysis of these two cluster randomized trials in the EHRs of CPRD. We report on issues that were common to both trials, as well as drawing attention to differences of approach where appropriate. Issues, such as missing data, which are also relevant to observational analyses of EHR data are not discussed in detail.

## Methods

The paper draws on the authors’ experience, documentary records of the trial, and trial datasets to report on the main practical issues involved in designing and performing a cluster randomized trial using EHRs. The paper begins with a brief outline of the purpose and design of the trials. It goes on to discuss ethical issues and research governance procedures, general practice recruitment and allocation, and sample size and power, drawing on data from the trial datasets. The following section discusses how the problem of intervention delivery was addressed. The final main section of the paper draws attention to some issues relevant for data analysis. The paper concludes with a brief discussion. Statistical analyses were performed in Stata version 13 Stata Corporation, College Station, Texas. Intraclass correlation coefficients were estimated using one way analysis of variance. The protocol for the research was approved by the MHRA Independent Scientific Advisory Committee (ISAC, protocol number 08_083) and the London-Surrey Borders NHS Research Ethics Committee (09-H0806-81 and 10-H0806-1).

## Results and discussion

### Outline of trial design

The stroke trial and the antibiotic trial shared similar objectives and design features while differing in the topic of application and approach to data analysis. The primary purpose of the research was to develop methods to perform cluster randomized trials using EHRs but the substantive topic of the trials was to facilitate translation of research evidence into practice in the areas of antibiotic prescribing and stroke secondary prevention. Each trial was a two-arm cluster randomized trial with general practice as the unit of allocation. In each trial, the active intervention consisted of a set of educational and decision support tools that were remotely installed into the software system of participating general practices and activated during consultations with eligible participants. General practices in the control trial arm continued with usual clinical practice. The development of the interventions [12] and a process evaluation of the intervention implementation (paper submitted for publication) have been reported elsewhere. In the antibiotic trial, eligible participants were those consulting for acute respiratory infections with the intervention aiming to reduce unnecessary antibiotic prescribing [13], following recommendations by the UK National Institute for Health and Care Excellence (NICE) [14]. In the stroke trial, participants were eligible for the intervention if they were included in the practice stroke register. The intervention, which was activated during any consultation by eligible patients, aimed to promote adherence with nationally recommended standards of care for stroke [15]. There was a 12-month intervention period in both trials. Data to evaluate participants’ baseline characteristics and trial outcomes were drawn from data routinely recorded into CPRD during consultations in primary care. In the antibiotic trial, a cluster level analysis was performed using practice-specific rates of consultation and antibiotic prescribing as observations, with the proportion of consultations with antibiotics prescribed as the primary outcome. In the stroke trial, an individual level analysis was performed on systolic blood pressure as the primary outcome, with marginal models estimated using generalized estimating equations.

### Ethical issues and research governance

The arrangements for research ethical approval and research governance for CPRD trials differ from other trials both because general practices contributing to CPRD are widely dispersed geographically and because general practices contribute to CPRD on an anonymized basis and it is not possible for research teams to contact them directly. Our experience may be relevant to the conduct of future trials in CPRD and similar data resources.

The protocol for the research was submitted to the Independent Scientific Advisory Committee, which is responsible for reviewing all proposed research in CPRD. The proposal was approved with minor revisions. The protocol for each trial was submitted to and approved by a local NHS Research Ethics Committee. Consent to participation in the study was requested from a senior partner at eligible CPRD general practices. The rationale for consent at the cluster level was that the intervention was implemented for the whole cluster by installing the intervention into the general practice software system with the practice staff being the intended recipients of the intervention [16]. Individual patient health record data were to be analysed to evaluate trial outcomes but the ethical issues associated with this data collection and analysis are covered by the overarching governance framework of CPRD. Weijer *et al.*[17] argue that in trials of the present type, individual patients should not be regarded as research participants because all treatment decisions remain the responsibility of the health professionals and are not determined by the trial allocation.

CPRD general practices participate in the database on the basis of anonymity. For this reason, all communications with practices were through CPRD and the trial research team did not have any direct contact with the trial practices. However, the consent form for the study included explicit consent for the practice to be identified to the intervention provider in order to allow activation of the intervention as outlined below in the event that the practice was allocated to the intervention trial arm. The consent form also included an item that requested permission for the practice to be contacted by the research team for a qualitative interview for the process evaluation of the intervention.

In the UK, research governance approval is also required from each participating locality-based NHS organisation. This presents a difficulty for CPRD research because general practices participating in CPRD are distributed throughout the UK, including England, Scotland, Wales, and Northern Ireland, with each territory having its own independent governance framework. As the location of CPRD practices is not generally made available to researchers, we aimed to obtain approvals from all NHS primary care organizations in England and Scotland for the antibiotic trial, and England, Scotland, and Wales for the stroke trial. Northern Ireland was not included in either trial as it is geographically more remote, but it might be feasible to include it in future studies. In England and Scotland, approvals were obtained through a system known as the central system for permissions (CSP) and NHS Research Scotland Permissions Coordinating Centre (NRSPCC), which facilitated the approval process at each local primary care organisation or health board (Scotland). In Wales, approvals were obtained from each health board. Table 1 presents data for the approvals obtained in England and Scotland for the antibiotic trial and England, Scotland, and Wales for the stroke trial. The majority of NHS organizations approved the trials, with 159 primary care organizations approving the antibiotic trial and 158 approving the stroke trial. However, a number of organizations declined to participate. In every case this was because the trial interventions were perceived to conflict with locally developed advice for general practice prescribing.

**Table 1:** Governance approvals from UK primary care organizations for the two trials

	**Invited**	**Approved**	**Declined**
**Antibiotic trial**			
PCTs in England	159	149	10
Health Boards in Scotland	10	10	0
**Stroke trial**			
PCTs in England	158	141	17
Health Boards in Scotland	12	10	2
Health Boards in Wales	7	7	0

Figures are numbers of primary care organizations. PCT, primary care trust.

### General practice recruitment and allocation

The recruitment process is critical to the success of most trials. In order to deliver recruitment for these studies, general practices participating in CPRD in areas for which research governance approvals were obtained were sent an invitation pack including an invitation letter, information sheet, and consent form. Table 2 shows the rate of recruitment to each trial. One reminder letter was sent to non-responding practices about two months after the initial invitation letter. In each trial, the recruitment target of 100 practices was exceeded within six months of the initial invitation letter.

**Table 2:** Recruitment of general practices into the two trials

	**Weeks from first invitation letter**	**Event**	**Cumulative number of general practices allocated**
**Antibiotic trial**	0	First invitation, England	0
	5	34 practices allocated	34
	7	Reminder, England; first invitation Scotland	34
	13	37 practices allocated	71
	16	Reminder, Scotland	71
	19	19 practices allocated	90
	23	11 practices allocated	101
	27	3 practices allocated	104
**Stroke trial**	0	First invitation	0
	5	71 practices allocated	71
	14	28 practices allocated	99
	21	7 practices allocated	106

Allocation of individual units to trial arms is a key design feature that protects against bias. In these two trials anonymized identifiers, with linked data for region and list size as stratifiers, were passed to King’s College London for allocation by minimisation [18]. Anonymized practice identifiers were then returned to CPRD with the trial arm allocation attached. This information was then used to enable intervention activation at practices in the intervention trial arm. This procedure was considered to ensure adequate concealment throughout the allocation process.

### Power and sample size

Estimating the size of a study is important in most trials. For trials in CPRD, such calculations may be readily informed by previously collected data. Sample size calculations for each trial, which drew on previous CPRD data analyses by the research team, have been reported previously [8,9]. However, analysis of trial data provided information concerning variability in cluster size, the extent of variation between practices, and the correlations between measures before and after intervention that might be used to provide improved sample size calculations. Initial calculations did not include data for variability in cluster sizes in terms of numbers of eligible participants per practice [19]. Table 3 presents empirical data for the distribution of cluster sizes in data from the two trials.

**Table 3:** Variation in cluster sizes (number of eligible participants per general practice) in two cluster randomized trials in CPRD

**Trial**	**Estimate**
**Antibiotic trial**	
Participants	Registered adults aged 18 to 59 years
Cluster size (median, IQR)	5,246 (3,608 to 7,219)
Minimum cluster size	811
Maximum cluster size	16,984
Mean (SD) cluster size	5,588 (2,938)
CV of cluster sizes	0.53
**Stroke trial**	
Participants	Patients with prevalent stroke
Cluster size (median, IQR)	102 (60 to 148)
Minimum cluster size	19
Maximum cluster size	343
Mean (SD) cluster size	110 (62)
CV of cluster sizes	0.56

CV*,* Coefficient of variation; IQR*,* Interquartile range; SD*,* Standard deviation.

As expected the mean cluster size differed considerably between the two trials with 110 prevalent stroke patients per practice in the stroke trial, but 5,588 registered patients aged 18 to 59 years per practice in the antibiotic trial. The coefficient of variation for cluster sizes was remarkably similar between the two trials, being 0.56 in the stroke trial and 0.53 in the antibiotic trial. These estimates are close to the median value for the coefficient of variation of practice list size (0.56, interquartile range 0.49 to 0.64) for all primary care organizations in England [20]. Eldridge *et al.*[20] showed that the design effect for a study will be greater when cluster sizes are variable rather than when they are uniform, with the usual design effect:

$$\mathrm{DE}=1+\left[m-1\right]\rho$$

being replaced by DE = 1 + [(cv^2^ + 1). m - 1]*ρ* (Equation 2)

where DE is the design effect, m is the mean cluster size, ρ is the intraclass correlation coefficient of the outcome of interest, and cv is the coefficient of variation of the cluster sizes. The latter formula indicates that the estimated design effect is likely to be substantially higher when variation in cluster size is considered. In EHRs research mean cluster sizes may often be large, as observed in these two trials, potentially giving rise to substantial design effects.

The analysis of trial data also allowed us to estimate the extent of variation in trial outcomes between practices. Intraclass correlation coefficients (ICC) for outcomes of blood pressure and total serum cholesterol from the stroke trial are shown in Table 4. These values are similar to estimates that we reported for pretrial analyses for the period 2003 to 2006 [21]. Adams *et al.*[22] reported data from 31 cluster-based studies in primary care, their 1,039 ICC estimates gave a median ICC of 0.01 (interquartile range 0 to 0.032). In the present data, ICC values differed slightly between intervention and control trial arms. This apparent difference, which was evident both before and after intervention, is unexplained and might result from random error. This serves to draw attention to the variability of ICC estimates that may be obtained from a single data source. Equivalent data for the antibiotic trial are shown in Table 5. Here coefficients of variation for practice-specific rates, rather than intraclass correlation coefficients, are presented following the approach developed by Hayes and Bennett [23]. These also indicate considerable variation between practices, as we have described previously from a clinical perspective [24,25].

**Table 4:** Intraclass correlation coefficient (95% confidence interval) and correlation between pre- and post-intervention measures for the stroke trial

	**Intraclass correlation coefficient (ICC) (95% confidence interval)**		**Correlation between outcome measures before and after intervention**
	**Before Intervention**	**After Intervention**	
**Systolic blood pressure (mmHg)**			
All trial participants	0.026 (0.016 to 0.037)	0.022 (0.012 to 0.031)	0.43
Control trial arm	0.043 (0.021 to 0.066)	0.037 (0.018 to 0.057)	0.46
Intervention trial arm	0.010 (0.002 to 0.018)	0.008 (0.0005 to 0.015)	0.40
**Diastolic blood pressure (mmHg)**			
All trial participants	0.023 (0.014 to 0.033)	0.016 (0.009 to 0.024)	0.42
Control trial arm	0.029 (0.013 to 0.046)	0.020 (0.008 to 0.033)	0.44
Intervention trial arm	0.018 (0.007 to 0.029)	0.013 (0.004 to 0.022)	0.40
**Total serum cholesterol (mmol/L)**			
All trial participants	0.010 (0.004 to 0.016)	0.015 (0.007 to 0.022)	0.77
Control trial arm	0.015 (0.004 to 0.026)	0.019 (0.007 to 0.031)	0.76
Intervention trial arm	0.005 (0.000 to 0.012)	0.011 (0.003 to 0.020)	0.78

**Table 5:** Design parameters from the antibiotic trial

	**Coefficients of variation for general practice specific rates or proportions**		**Correlation between rates before and after intervention**
	**Before intervention**	**After intervention**	
**RTI consultation rate**			
All trial practices	0.27	0.26	0.83
Control trial arm	0.22	0.24	0.75
Intervention trial arm	0.31	0.28	0.89
**Antibiotic prescribing rate**			
All trial practices	0.35	0.36	0.82
Control trial arm	0.31	0.36	0.79
Intervention trial arm	0.38	0.35	0.88
**Proportion of consultations with antibiotic prescribed**			
All trial practices	0.20	0.20	0.91
Control trial arm	0.20	0.20	0.91
Intervention trial arm	0.20	0.20	0.91

Figures are coefficients of variation of practice-specific rates or proportions and correlation coefficients between the same measures before and after intervention. RTI, respiratory tract infection.

Tables 4 and 5 also present data for the correlation of outcomes between the 12 months before intervention and the 12 months after the start of intervention. In the stroke trial, outcomes of blood pressure and total cholesterol were found to be highly correlated in individual patient data, with correlation coefficients in excess of 0.4 for systolic and diastolic blood pressure and 0.7 for total cholesterol. In the antibiotic trial, correlations from before and after intervention were generally greater than 0.8 for rates on consultation for respiratory tract infection, rates of antibiotic prescribing, and the proportion of consultations with antibiotics prescribed. These correlations (r) show that, although there is substantial variation between practices and individuals, there is a considerable stability of values over time within practices or individuals. When trial analyses are performed in an analysis of covariance (ANCOVA) framework, these correlations may result in considerably more precise estimates than anticipated from sample size calculations that only considered differences between trial arms at the end of intervention [26]. The design effect appropriate for a post-test only analysis can be multiplied by 1-r^2^ to correct for a clustered ANCOVA design [26,27]. The stability of estimates over time implied by these correlations suggests that it may be worthwhile to construct elements of trial analysis in the primary care database in advance of the trial in order to obtain relevant design parameters to inform sample size calculations. However, in EHR research the marginal cost of increasing the numbers of clusters in a study might be small, depending on the costs of intervention.

### Intervention implementation

The purpose of intervention development and implementation was to deliver educational and decision support tools to general practitioners (GPs) at the point-of-care during routine consultations [12]. The intervention included evidence-based recommendations to GPs, external links to guidelines and research evidence to support clinical decision-making, as well as printable patient information. Identifying a method through which the intervention could be delivered was an important element of this project. CPRD general practices utilise a software system known as VISION. Initially, we considered utilising a bespoke program, which was to be developed in-house, to deliver the intervention. This approach was used to facilitate patient recruitment in the CPRD clinical trials RETROPRO and eLung which recruited from a much smaller number of CPRD practices [28]. However, this approach proved time-consuming and difficult. Instead, the intervention was delivered through a system known as DXS Point-of-Care [29] DXS UK Ltd, Farnham, UK, which is already integrated into VISION. The DXS Point of Care system delivered the intervention as a set of webpages with multiple external links. When practices were allocated to the intervention trial arm the intervention was activated by DXS Point-of-Care. An increasing number of CPRD practices use a version of VISION software that is hosted on an external shared server. Implementation of the intervention for these server-hosted practices proved more technically challenging and time consuming, but was achieved. An attractive feature of the DXS method was the collection of data on utilisation of the intervention. This enabled us to monitor GPs adherence to the intervention and relate study outcomes to uptake and utilisation of the intervention.

The intervention was activated through information recorded into patients’ EHRs during consultations. In the initial stages of the project it was only possible to utilize Read medical codes, recorded during the index consultation, to activate the intervention. In the antibiotic trial, which was completed first, the intervention was activated when a Read medical code for acute respiratory tract infection was entered during a participant’s consultation. Subsequently, access to a wider range of information from the patient’s EHR was facilitated. For the stroke trial, the intervention was activated during any consultation by a patient who was included in the practice stroke register. In the UK, general practices maintain registers of a number of chronic diseases as a part of their contractual obligations [30]. As part of this process the practice maintains a register of all patients registered with stroke or transient ischaemic attack. These were identified as participants who were eligible for the intervention. However, only patients with previous stroke were eligible to be included in the trial analysis. Patients with transient ischaemic attack were not included because this diagnosis may have poor specificity in routine clinical practice.

It is important to consider the behaviour of end-users in the design and implementation of the intervention. In these trials, communication of the intervention required that end-users should click on a link in order to read the intervention materials. This requires users to actively seek new information to inform clinical practice even in conditions that may be regarded as routine. Although we had the capability to deliver active alerting through the use of ‘pop-ups’, this approach was not used because of qualitative evidence that active alerts are annoying and off-putting to users. The intervention only became active when information was entered into the clinical record and for those general practitioners who only enter clinical data after the end of the consultation the opportunity to influence practice in that consultation might be lost. However, the educational tools might have a lasting effect on clinical practice after being viewed only once.

Our experience shows that it is feasible to introduce intervention materials into the software systems of CPRD general practices that are participating in a trial. Furthermore, it was possible to monitor the utilisation of the intervention and conduct a qualitative process evaluation to explore end-users’ experience of utilising the intervention materials. However, future trials in CPRD will need to develop a more diverse range of effective interventions so as to broaden the scope of future intervention studies.

### Analysis issues

Trials in CPRD benefit from the assessment of outcomes for large numbers of participants using data that are routinely collected in EHRs. The strengths and limitations of such data have been extensively considered elsewhere. The two trials adopted differing approaches to analysis with the stroke trial using an analysis of individual participant level data [31] and the antibiotic trial utilising a cluster level analysis of practice-specific rates and proportions weighted to allow for varying cluster sizes [20]. These represent standard methods of analysis. However, in a primary care database such as CPRD there is unusual flexibility in the selection of data for analysis, and this may be of considerable importance. In order to inform future research studies we first discuss the selection of stroke cases and eligibility criteria for the stroke trial. We then go on to consider issues of person time and the inclusion of general practices in the analysis.

### Individual participants and eligibility criteria

In primary care EHRs, cases are generally selected on the basis of Read medical codes. The Read code classification is partly hierarchical, drawing on disease categories that map to the International Classification of Diseases. However, there are also codes for symptoms, clinical signs, medical tests, and interventions among others. This results in a diverse range of codes being available to code a condition such as stroke. Typically, small numbers of codes are frequently used, while a broader range of codes may be used at intermediate or low frequency. We have previously reported on the use of medical diagnostic codes for stroke in CPRD [32], presenting a range of options for case definition of stroke. For the present study we used a stringent definition, including only codes that were considered to provide firm evidence of an acute stroke.

Even with a fixed-case definition there is a range of possibilities for including individual participants, as outlined in Table 6. The most inclusive option for the stroke trial was to include all participants with acute stroke ever recorded before the intervention start date. There were 11,391 participants at trial practices that met this criterion for prevalent stroke who survived to the start of intervention. A second option was to include participants with acute stroke recorded as an incident event more than 12 months after the start of the current registration in CPRD. This criterion excluded participants with prevalent stroke diagnoses from before the start of the ‘up-to standard’ CPRD record for whom the initial diagnoses might be less secure. There were 6,296 participants with incident strokes at trial practices (Table 6). A third option, which was initially considered in the trial protocol [9], was to include only participants with acute stroke within two years of the trial intervention start date. The rationale for this criterion was that GPs might be more amenable to modifying the management of participants with recent strokes. There were 1,706 participants at trial practices with incident strokes in the two years before the intervention start date. Table 6 presents selected baseline characteristics according to these three eligibility criteria for trial practices and for participants at non-trial CPRD practices. In this trial, participant characteristics were similar after selection according to either criterion, although participants with more recent strokes tended to have slightly higher blood pressure and total cholesterol values. Participant characteristics were also similar for trial and non-trial practices. The major impact of varying the inclusion criteria was on the sample size available for analysis. It is possible that, in a different trial, varying the inclusion criteria in this way might have a substantial impact on participant characteristics and estimated intervention effects. Ideally, pretrial analyses in CPRD would be sufficient to develop clearly defined eligibility criteria. Additionally, it is desirable to perform a sensitivity analysis to explore the effect of varying trial inclusion criteria.

**Table 6:** **Illustrating the effect on sample size and participant characteristics at baseline of varying participant selection criteria in the stroke trial.** CPRD, Clinical Practice Research Datalink.

	**Number**	**Age**	**Gender**	**Systolic blood pressure**	**Total cholesterol**
		**Mean (SD)**	**Female (%)**	**Mean (SD)**	**Mean (SD)**
**Participants with prevalent stroke**					
All Non-trial CPRD practices	47,887	72.2 (14.0)	23,179 (48)	134.4 (15.3)	4.4 (1.0)
Trial practices	11,391	72.5 (14.0)	5,490 (48)	134.5 (15.6)	4.4 (1.0)
**Participants with incident stroke since start of CPRD record**					
All Non-trial CPRD practices	27,971	72.5 (13.4)	13,369 (48)	134.8 (15.1)	4.4 (1.0)
Trial practices	6,296	72.5 (13.4)	2,950 (47)	135.0 (15.4)	4.4 (1.0)
**Participants with incident stroke within two years of intervention start date**					
All Non-trial CPRD practices	7,530	71.4 (14.0)	3,629 (48)	136.6 (15.6)	4.5 (1.10)
Trial practices	1,706	72.0 (14.1)	797 (47)	136.3 (16.5)	4.5 (1.10)

### Person time-at-risk and practices with no participants

Most CPRD studies employ longitudinal data analysis based on person time-at-risk. For CPRD practices, time-at-risk begins at the practice’s UTS start date and ends at the last data collection date. The latter reflects the most recent data collection from the practice but this may also indicate when the practice left the CPRD. For individual participants, time-at-risk starts at the date of their current registration (if this is after the practice’s UTS start date) and ends at the end of the registration or death (if these are before the practice’s last data collection date). Table 7 shows the time from UTS start to intervention start for trial practices. The median duration of participation in CPRD before the start of the trial was approximately 12 years for both the stroke trial and the antibiotic trial. In the stroke trial, the UTS date was before the intervention start date for all practices. However, in the antibiotic trial the UTS start date was found to be after the intervention start date for three practices, two in the intervention trial arm and one in the control trial arm. These practices were omitted from the analysis because participant data were only eligible from the UTS start date and pre-intervention as well as post-intervention observations were required for analysis. Table 7 also shows the median interval from intervention start to last data collection date; this was more than one year as intended. However, there were two control practices in the stroke trial and one intervention practice in the antibiotic trial with a last collection date that was before the intervention start date. These practices were omitted from the analysis. There were a further number of practices for which the last data collection date fell before the end of 12 months after the intervention. These practices’ data were analysed on the basis of person time-at-risk. These observations point to the importance of considering practices eligibility for analysis over time in relation to the implementation of the intervention.

**Table 7:** General practice eligibility for analysis in the two trials

	**UTS start to intervention start (median IQR, Years)**	**Number of practices with UTS start > intervention start**	**Intervention start to last collection date (median IQR, Years)**	**Number of practices with last collection before intervention start**	**Number of practices with last collection before intervention start plus one year**
**Antibiotic trial**					
Intervention practices	11.9 (8.6 to 14.9)	2	1.6 (1.4 to 1.7)	1	1
Control practices	12.1 (9.3 to 17.5)	1	1.5 (1.4 to 1.7)	0	4
**Stroke trial**					
Intervention practices	12.4 (9.9 to 19.9)	0	1.3 (1.2 to 1.4)	0	2
Control practices	13.7 (11.2 to 21.5)	0	1.4 (1.3 to 1.5)	2	3

IQR, interquartile range; UTS, up-to-standard.

## Conclusions

There is great interest in conducting clinical trials using EHRs but few trials have yet been completed. Our experience of completing two cluster randomized trials has identified several issues of methodological importance. The governance of a trial using EHRs may represent a time-consuming and challenging process, and this needs to be taken into account at the planning stage. The design and analytical approaches to be employed in a trial using EHRs should carefully consider case definitions, eligibility criteria for practices, and individual participants. The definition, recording, and variability of outcome measures also require consideration. These issues may often be addressed through pretrial analysis of EHR data. Future studies should aim to increase the scope and complexity of interventions that can be delivered in EHR-based trials with attention to behavioural considerations that may influence uptake and effectiveness.

## Abbreviations

ANCOVA: Analysis of covariance; CPRD: Clinical Practice Research Datalink; CSP: Central system for permissions; cv: Coefficient of variation; DE: Design effect; EHR: Electronic health record; GP: General practice; ICC: Intraclass correlation coefficient; NHS: National Health Service; NRSPCC: NHS Research Scotland Permissions Coordinating Centre; r: Correlation coefficient; UTS: Up-to-standard; UK: United Kingdom.

## Competing interests

The authors declare that they have no competing interests.

## Authors’ contributions

MG designed and supervised the project. LM designed and developed the trial interventions. JC analyzed the CPRD data. AD contributed research governance approvals, general practice recruitment and allocation, and data analysis. TvS contributed to trial design and general practice recruitment. GM was responsible for CPRD general practice recruitment and liaison during the trials. MG drafted the paper. All authors contributed to the paper and approved the final draft. All authors read and approved the final manuscript.
